# Using the SUBcellular database for *Arabidopsis* proteins to localize the Deg protease family

**DOI:** 10.3389/fpls.2014.00396

**Published:** 2014-08-12

**Authors:** Sandra K. Tanz, Ian Castleden, Cornelia M. Hooper, Ian Small, A. Harvey Millar

**Affiliations:** ^1^The Australian Research Council Centre of Excellence in Plant Energy Biology, The University of Western AustraliaPerth, WA, Australia; ^2^Centre of Excellence in Computational Systems Biology, The University of Western AustraliaPerth, WA, Australia; ^3^Centre for Comparative Analysis on Biomolecular Networks, The University of Western AustraliaPerth, WA, Australia

**Keywords:** subcellular localization, database, *Arabidopsis*, protein, Deg protease

## Abstract

Sub-functionalization during the expansion of gene families in eukaryotes has occurred in part through specific subcellular localization of different family members. To better understand this process in plants, compiled records of large-scale proteomic and fluorescent protein localization datasets can be explored and bioinformatic predictions for protein localization can be used to predict the gaps in experimental data. This process can be followed by targeted experiments to test predictions. The SUBA3 database is a free web-service at http://suba.plantenergy.uwa.edu.au that helps users to explore reported experimental data and predictions concerning proteins encoded by gene families and to define the experiments required to locate these homologous sets of proteins. Here we show how SUBA3 can be used to explore the subcellular location of the Deg protease family of ATP-independent serine endopeptidases (Deg1–Deg16). Combined data integration and new experiments refined location information for Deg1 and Deg9, confirmed Deg2, Deg5, and Deg8 in plastids and Deg 15 in peroxisomes and provide substantial experimental evidence for mitochondrial localized Deg proteases. Two of these, Deg3 and Deg10, additionally localized to the plastid, revealing novel dual-targeted Deg proteases in the plastid and the mitochondrion. SUBA3 is continually updated to ensure that researchers can use the latest published data when planning the experimental steps remaining to localize gene family functions.

## INTRODUCTION

The expansion of gene families in eukaryotes has divided function between members in a variety of ways (Massingham et al., 2001; Rutter et al., 2012; Wang et al., 2012). One such division has been the diversification of localization of protein family members to different parts of the cell (e.g., for isoprenoid metabolism; Beck et al., 2013). Interconnected metabolic and regulatory pathways operate in distinct subcellular compartments and the proteins that perform these processes are restricted by compartment boundaries. An important step toward defining the biochemical role of a given protein family member is therefore to identify the intracellular location in which it accumulates and functions.

Researchers can determine the subcellular location of proteins by a number of approaches. These include *in silico* prediction methods and experimental approaches. Computational prediction programs are often based on machine-learning algorithms that search for sequence features in a primary amino acid sequence to predict the likelihood that a protein is found in a specific subcellular location. These computer programs have become critical tools for annotating newly sequenced genomes on a large scale. Experimental approaches that are available for confirming subcellular location include *in vitro* protein import studies into isolated organelles, *in vivo* protein tagging by fluorescent markers, enzyme activity measurements, immunolocalization, or cell fractionation followed by protein detection using mass spectrometry (Millar et al., 2009). It is important to note that localization data sets obtained from such experiments form the basis of both the determination of subcellular localization and the set up of training sets that are used to create prediction programs.

Proteomic studies employ mass spectrometry to identify proteins in enriched subcellular compartments and lead to large, information-rich datasets. Purification techniques have improved rapidly over the last decade and have allowed better identification of more specific subcellular locations. For example, the combination of density gradient centrifugation with free-flow electrophoresis was employed to improve the separation of tonoplast from plasma membranes (Bardy et al., 1998), mitochondria from peroxisomes and plastids (Eubel et al., 2007), and the isolation of Golgi membranes (Parsons et al., 2012). In addition, novel analysis strategies have been developed, such as intelligent data-dependent acquisition (IDDA), that can increase the number of peptide ions analyzed in the mass spectrometer and consequently improve the identification of peptides and proteins relative to previous methods (Eubel et al., 2008; Hoopmann et al., 2009).

Another experimental approach that is widely used to localize proteins in the cell is the expression and visualization of fluorescent proteins (FPs) that are attached to the proteins of interest. Notably, *in vivo* FP tagging is the only subcellular location method that provides data for intact, living cells. However, the positioning of the FP in a chimeric construct is important as it can mask the targeting ability of a protein signal peptide and this can greatly affect the accuracy of the localization results. For example, an *N*-terminally tagged mitochondrial protein is likely to be mistargeted and so is a *C*-terminally tagged peroxisomal protein. Often this method is referred to as green FP (GFP) tagging because GFP is one of the most frequently used FPs (Chiu et al., 1996). Thousands of *Arabidopsis* proteins have been visualized using this direct approach (including some high-throughput GFP screens) and these form an important resource for determining subcellular location (Tian et al., 2004; Koroleva et al., 2005; Li et al., 2006; Carrie et al., 2009; Van Aken et al., 2009; Boruc et al., 2010; Lee et al., 2011; Narsai et al., 2011; Inze et al., 2012).

Predicted and experimental localisation data are scattered in the literature and researchers can spend large amounts of time and effort to ensure all published localization information for a given protein has been collated. In fact, despite best efforts, published data can easily be overlooked as large number of protein localizations can be reported in an article but not listed in the title, abstract or text. In addition, curated subcellular proteomes and catalogs of GFP targeting information are not readily available as defined data sets for specific cellular locations.

The SUBcellular localization database for *Arabidopsis* proteins (SUBA; Heazlewood et al., 2005, 2007; Tanz et al., 2013) aggregates these datasets to combine prediction of protein localization for *Arabidopsis* proteins with experimental data and annotations. SUBA3 also includes a naive Bayesian classifier (SUBAcon) to provide a likely consensus location of a protein within the cell (Tanz et al., 2013). SUBA has previously been used for assessing targeting prediction programs (Heazlewood et al., 2004; Ryngajllo et al., 2011), for building metabolic network models (de Oliveira Dal’Molin et al., 2010; Mintz-Oron et al., 2012), and for analyzing co-expression and protein–protein interaction (PPI) data (Cui et al., 2011; Ryngajllo et al., 2011). Here we highlight features of SUBA3 that can be used to explore protein families by using the Deg protease family in *Arabidopsis* as an example. The Deg protease family was chosen because experimental localization data for some members of this family were complex, including conflicting data and the absence of any experimental data for a range of family members. This analysis is used for prioritizing and performing experiments highlighted by SUBA3, which were required to complete the localization of this protein family.

## MATERIAL AND METHODS

### SUBA3 DATABASE AND DATA SOURCES

SUBA3 can easily be queried through a web-browser based graphical user interface (GUI) that is freely available at http://suba.plantenergy.uwa.edu.au. The interface works best via the Mozilla Firefox, Google Chrome, or Safari web browsers but will work on Microsoft Explorer (6 and above). Currently, 24,142 entries are based on subcellular proteomic studies (7891 distinct proteins), 4110 entries are based on FP tagging studies (2647 distinct proteins), and 13,164 entries are based on PPI studies (4999 distinct proteins). SUBA3 also contains bioinformatic predictions for protein localization from the output of 22 prediction programs. Details of the database structure and sources have been described previously (Tanz et al., 2013). To best estimate a protein’s location in the cell, SUBA3 also contains a consensus location (SUBAcon) based on Bayesian probabilities calculated from all the experimental localization data and predictions available for each protein.

### SUBCELLULAR LOCALIZATION BY GFP TAGGING

The full-length coding sequences where ever possible, or the first 222–300 bp of the coding sequences of *Deg1–Deg16* were amplified according to the manufacturer’s instructions using the Expand High Fidelity PCR system (Roche Diagnostics) with primers listed in Supplemental Table 1 containing the *attB* sites for Gateway^®^ cloning. The PCR products of *Deg1–Deg15* were cloned into the Gateway^®^ vector pDONR207 (Invitrogen) and sequenced. The entry clone and a Gateway^®^ cloning cassette (pDest/pgem/CGFP; Carrie et al., 2009) were recombined to clone the full-length or the first 222–300 bp in frame with the coding region of the GFP at their *N*- or *C*-terminus. For co-localization studies, the small subunit (SSU) of *Arabidopsis* ribulose-1,5-bisphosphate carboxylase oxygenase fused to the *N*-terminus of the red FP (SSU-RFP; Carrie et al., 2009) was used as a plastid control, the mitochondrial targeting sequence of yeast *ScCox4* fused to the *N*-terminus of mCherry in pBIN20 (mt-rk *CD3-991*; Nelson et al., 2007) was used as a mitochondrial control, and the peroxisomal targeting signal 1 (PTS1, Ser-Lys-Leu) fused to the *C*-terminus of mCherry (px-rk *CD3-983*; Nelson et al., 2007) was used as peroxisome control. The fusion constructs were biolistically transformed into cultured *Arabidopsis* cells. The GFP and RFP/mCherry plasmids (5 μg of each) were co-precipitated onto 1-μm gold particles and transformed using the biolistic PDS-1000/He system (Bio-Rad). Particles were bombarded onto 2 mL of cultured *Arabidopsis* cells resting on filter paper on osmoticum plates (2.17 g/L Murashige and Skoog Modified Basal Salt Mixture, 30 g/L sucrose, 0.5 mg/L naphthalene acetic acid, 0.05 mg/L kinetin, 36.44 g/L mannitol). After bombardment, the cells were placed in the dark at 22°C. Fluorescence images were obtained 24 h after transformation using an Olympus BX61 epifluorescence microscope with excitation wavelengths of 460/480 nm (GFP) and 535/555 nm (RFP and mCherry), and emission wavelengths of 495–540 nm (GFP) and 570–625 nm (RFP and mCherry). Subsequent images were captured using Cell^®^ imaging software.

## RESULTS

### QUERYING SUBA3 TO LOCATE DATA ON MEMBERS OF PROTEIN FAMILIES

**FIGURE 1:**
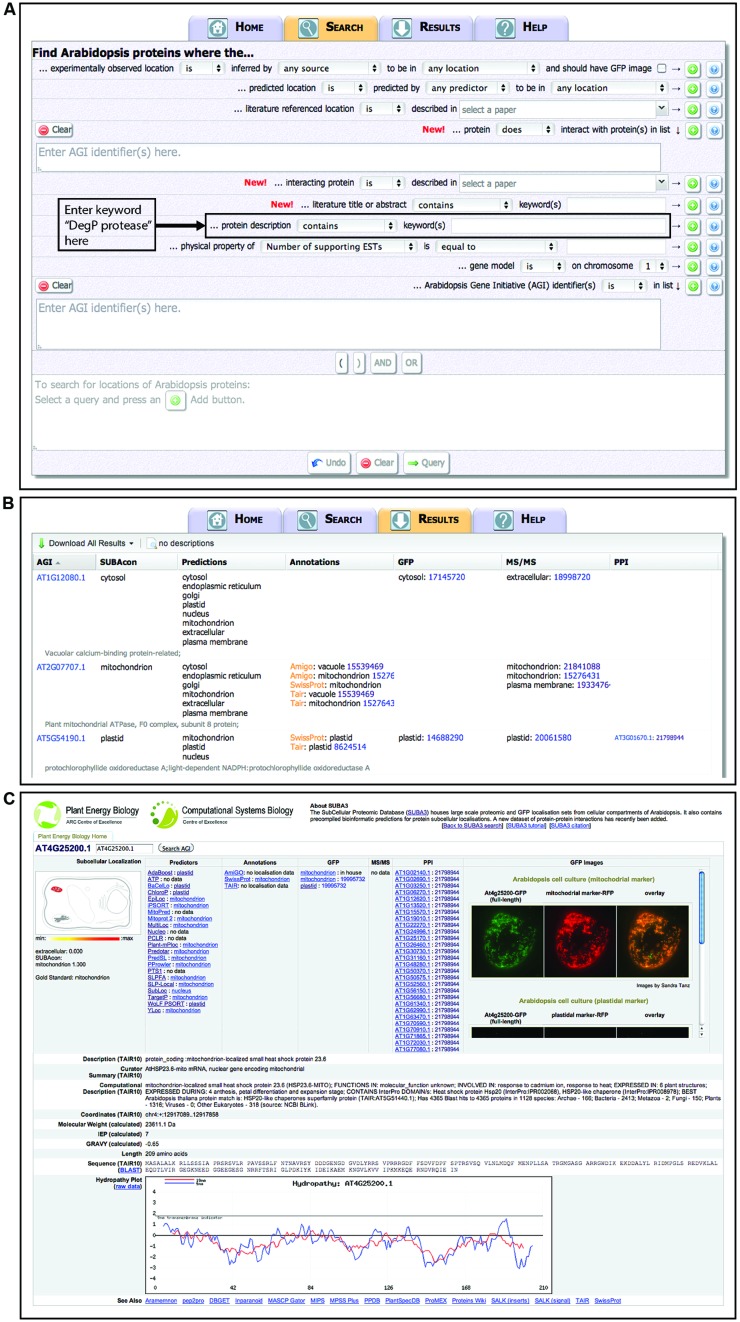
**The SUBA3 interface.** Screenshots showing the SUBA3 search page **(A)** where complex queries can be build using pull down menus in combination with AND, OR, and bracketing functions. The SUBA3 Results page **(B)** of a query is shown with seven default columns and a “Download All Results” button in the left top corner. An example of a SUBA3 flatfile **(C)** showing detailed localization information of predictions, annotations, and experimental data (GFP, mass spectrometry, protein–protein interaction data), the SUBAcon call displayed as pictographic heat map of a plant cell, GFP images, and other details such as the description, amino acid sequence, hydropathy plot and links to other useful websites at the bottom of the page.

Users can operate the SUBA3 interface^1^ to ask simple questions about one protein at a time or to construct moderately complex SQL queries using drop down menus and buttons to perform powerful Boolean queries (AND, OR, NOT) across all entries for a protein family. The primary “Search” tab allows the development of the query (**Figure 1A**). Once a query has been submitted, the “Results” page shows a table, which contains the *Arabidopsis* genome initiative (AGI) identifier, a short description, summary localization information from SUBAcon, predictions, annotations, GFP, mass spectrometry, and PPI data (**Figure 1B**). Nearly all retrieved data are linked to a reference in PubMed^2^. A variety of information and helpful links for each AGI in the result page is hyperlinked by access to the “SUBA flatfile” (**Figure 1C**). These data include detailed information on the subcellular localization predictions, a cell cartoon displaying the probability values of the consensus location (SUBAcon), access to GFP images for localizations if available, and links to other resources for this AGI (**Figure 1C**).

To query a particular gene family, the full list of AGI identifiers can be entered in the “AGI” input box and directly queried in SUBA3. Alternatively, SUBA3 can be searched by entering a descriptor in the “keyword” input box, such as the name of a particular gene family in the TAIR10 genome annotation. For many gene families, this type of search can be used to rapidly assess the predicted location and, if applicable, collate the experimental location information for each member. The importance of multiple pieces of independent evidence for high confidence assessments of subcellular location has been highlighted (Millar et al., 2009). SUBA3 rapidly provides clarity on the available evidence and the methods used, allowing researchers to make decisions to augment what is currently known with independent experimental approaches.

**FIGURE 2:**
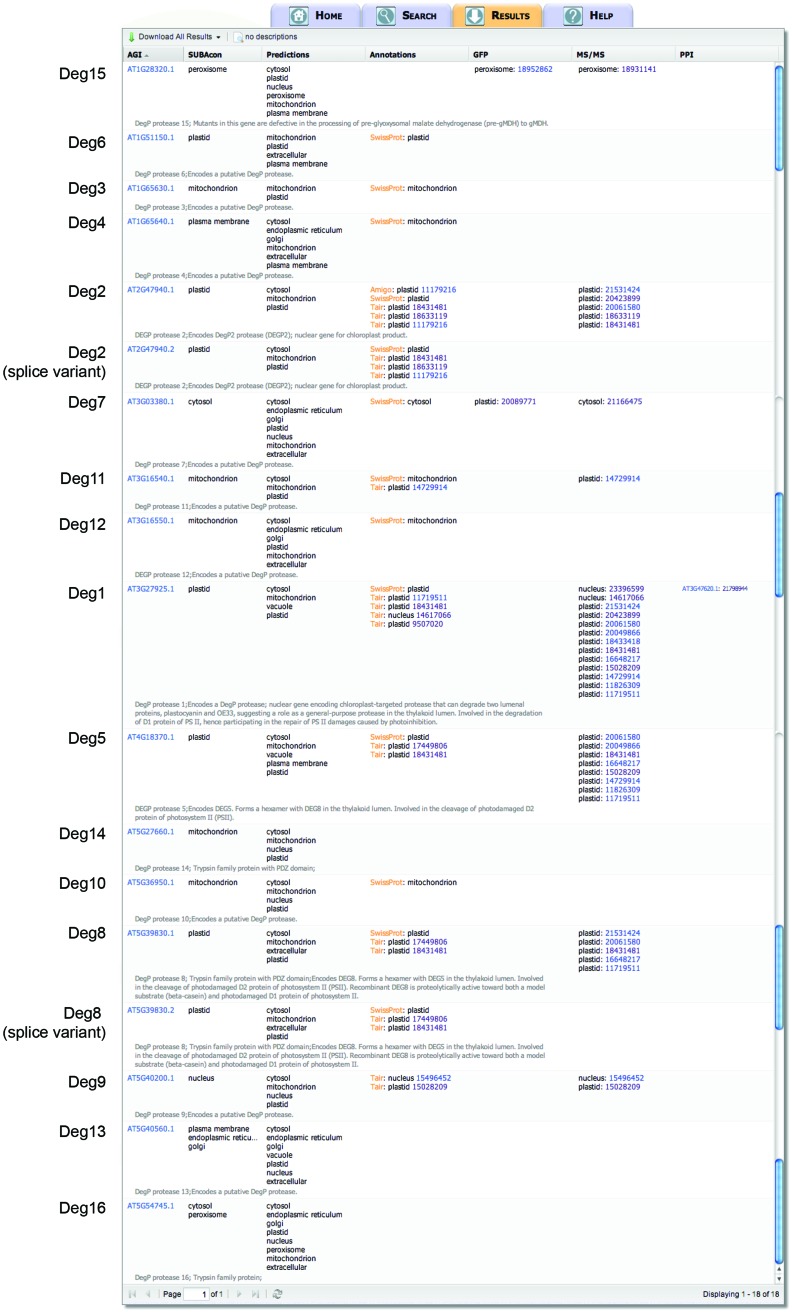
**SUBA3 result output for Deg proteases in *Arabidopsis*.** Querying the SUBA3 database by entering “DegP protease” as descriptor in the “keyword” input box returns results for 18 gene products with location evidence. Displayed are the location calls by SUBAcon, the predicted locations determined by the 22 predictors in SUBA3 (column “Predictions”), the locations from the annotators TAIR, AmiGO, and UniProt/SwissProt (column “Annotations”), the experimentally determined locations by GFP tagging and mass spectrometry, as well as protein–protein interaction (PPI) data. Short TAIR descriptions are displayed in gray and the name of each Deg family member is indicated on the left.

Using the search term “DegP protease” as a keyword in a SUBA3 search (as indicated in **Figure 1A**) yields 18 proteins (**Figure 2**). Note, the nomenclature of “DegP proteases” has recently changed and we will now refer to them collectively as “Deg proteases” (Huesgen et al., 2005). This family of ATP-independent serine endopeptidases (originally named for “degradation of periplasmic proteins”) functions in various proteolytic events in the cell. Sixteen different Deg protease gene loci are known in *Arabidopsis* (Deg1–Deg16). Two of the 18 result outputs from SUBA3, At2g47940.2 and At5g39830.2, are alternative splice variants of Deg2 and Deg8, respectively (**Figure 2**). Examining the SUBAcon results for the remaining 16 Deg proteases shows that they are predicted to eight of the 11 different physical locations in SUBA3 (namely cytosol, endoplasmic reticulum, Golgi, mitochondrion, nucleus, peroxisome, plasma membrane, and plastid). Eight Deg proteases have been identified in subcellular proteomic studies, and two of those eight have additionally been localized by GFP tagging (**Figure 2**). Studying these data shows there are agreements and disagreements between predictors and/or between experimental datasets for the location of Deg proteases. This raises a series of issues that can be addressed in turn by further analysis in SUBA. Firstly, when experimental data are absent but prediction is clear, is the experimental coverage of a given location extensive enough to expect the protein to have been found? Secondly, when experimental data disagree could specific experiments be erroneous? Thirdly, when experimental data disagree, could the protein be multi-targeted?

### DEFINING THE SIZE OF EXPERIMENTAL VS PREDICTED DATASETS FOR A SPECIFIC SUBCELLULAR LOCATION

To determine in which subcellular location a protein without experimental localization data can be expected to be found, it is often very beneficial to generate a list of the subcellular locations of proteins by different experimental methods (**Table 1**). An additional step supported by SUBA is combining experimental sets like mass spectrometry and GFP tagging (“MS/MS assay” OR “GFP assay”). Using this approach, plastid and mitochondrial sets can be defined that contain 2385 and 1034 proteins respectively. These sets can be expanded by adding other proteins that are predicted to be in the relevant organelle to construct the total predicted proteome. For example, by including proteins predicted by the three predictors TargetP, Predotar and YLoc [“MS/MS assay” OR “GFP assay” OR (“TargetP” AND “Predotar” AND “YLoc”)], the plastid proteome set is 3026 proteins and the mitochondrial set is 1651. The sizes of a variety of organelle proteomes in plants and other eukaryotes have been previously reported in the literature (**Table 1**).

**Table 1:** Compiled localization data collected in SUBA3.

Location	GFP	MS	Distinct proteins localized by GFP or MS	Reported subcellular proteome size
**Cytoskeleton**	68	0	68	–
**Cytosol**	580	1808	2262	4000^1^
**Endoplasmic reticulum**	202	315	469	–
**Extracellular**	45	471	507	–
**Golgi**	176	720	832	–
**Mitochondrion**	318	815	1034	2000^2^
**Nucleus**	845	897	1610	7800^1^
**Peroxisome**	139	204	280	670^3^
**Plasma membrane**	275	3863	4006	–
**Plastid**	510	2133	2385	4000^4^
**Vacuole**	103	786	851	–
**Unclear**	178	132	302	–
**Any location**	2647	7891	9319	–

*Numbers represent distinct *Arabidopsis* proteins that are located in 11 subcellular compartments and a 12th category referred to unclear, for which location data are considered speculative by the authors of the report. Also listed are estimated proteome sizes for five subcellular compartments of *Arabidopsis*: cytosol, mitochondrion, nucleus, peroxisome, and plastid. GFP, data from fluorescent protein tagging experiments; MS, data from mass spectrometry analysis of isolated subcellular compartments.*

*^1^Guda (2010; based on estimation from other eukaryotes).*

*^2^Millar et al. (2006).*

*^3^Bussell et al. (2013).*

*^4^Martin et al. (2002) and Kleffmann et al. (2004).*

**Table 1** shows that over 2000 proteins have been located to the plastid by MS analysis, which represents almost a third of all proteins located by this method. Similarly, proteins located to the plastid by GFP tagging represent almost a fifth of all the proteins located by GFP tagging. Taken together, these datasets give a 60% experimental coverage of the estimated plastid proteome size in *Arabidopsis* (**Table 1**). Thus, it can be expected that experimental data are likely to exist for Deg proteases located in plastids. In fact, seven of the eight Deg proteases, for which experimental data exist, have been localized to the plastid by the MS or GFP approach (**Figure 2**). In comparison, only one Deg protease has been localized to the peroxisome. Significantly fewer proteins have been localized to the peroxisome experimentally and the coverage of the estimated peroxisome proteome is only 42% (**Table 1**) leaving more room for unidentified Deg proteases to be found in this location.

### CONFLICTS BETWEEN PUBLISHED LOCALIZATION DATASETS

As new reports of subcellular locations of proteins accumulate in the literature discrepancies with previous observations inevitably accumulate as well. SUBA3 gives an overview of these data sets and by using the “Literature referenced location is/is not described in …” option on the “Search” page, SUBA3 gives access to data sets from each individual paper. To directly compare these individual data sets and to determine whether claimed locations have also been reported by other groups, the AND, OR, and bracketing connectors in the “Search” window can be used. For example, Reumann et al. (2007) and Eubel et al. (2008) began to define the peroxisomal proteome by mass spectrometry and GFP analysis, listing 79 and 115 proteins, respectively. The common set between the two studies is 53 (Reumann et al., 2007 AND Eubel et al., 2008). In 2009, Reumann et al. published a larger protein set of 151 peroxisomal proteins and the common set between the two groups rose to 73 proteins [(Reumann et al., 2007 OR Reumann et al., 2009) AND Eubel et al., 2008]. Similarly, Ferro et al. (2010) and Olinares et al. (2010) published plastid proteome sets of 1321 and 586, respectively. The common set of 473 proteins shows that 81% of the proteins listed in Olinares et al. (2010) were also found by Ferro et al. (2010).

When a conflict between experimental data exists for a gene family (e.g., as seen in **Figure 2** for Deg1, Deg7, and Deg9), SUBA3 users can directly compare the individual research publications that reported the data sets and determine whether similar conflicts/contaminations have also been reported by other groups. Deg9, for example, has been localized by MS to two different locations by two independent research groups. Pendle et al. (2005) found Deg9 in the nucleus, whereas Kleffmann et al. (2004) claim a plastid location for this protein. Using the “Literature reference location” search with the AND connector (Pendle et al., 2005 AND Kleffmann et al., 2004), results in a shared set of 61 proteins that are claimed to be located in both compartments, the nucleus and plastid. Examining this set of 61 proteins further, showed that 20 of the 26 proteins for which independent GFP tagging data exist were confirmed to be located in the nucleus, whereas a plastid location could only be verified for two proteins. This suggests that a nuclear location for Deg9 is more likely than a plastid location.

### POTENTIALLY MULTI-TARGETED PROTEINS

Multi-targeting of proteins was originally expected to be a rare event, given the specialized function of the different subcellular compartments in a cell. However, the number of proteins that have been shown to be multi-targeted has greatly increased. Currently, more than 100 proteins are known to be dual-targeted to mitochondria and plastids in *Arabidopsis* (Carrie and Small, 2013). This is due to the ease by which dual-targeting can be confirmed by GFP tagging and because of an increased interest in processes expected to be common to these two organelles. These processes include DNA replication and repair, transcription, translation, and proteolysis. Many proteins have been found to be dual-targeted between other organelles in plants, including mitochondria, plastids and cytosol (Small et al., 1998), mitochondrion, plastid and endoplasmic reticulum (Lee et al., 2011), nucleus and cytosol (Inze et al., 2012), plastids and nucleus (Schwacke et al., 2007), mitochondria and nucleus (Krause and Krupinska, 2009), plastids and peroxisomes (Reumann et al., 2007; Sapir-Mir et al., 2008), mitochondria and peroxisomes (Carrie et al., 2008, 2009), plastid and cytosol (Kiessling et al., 2004; Thatcher et al., 2007), mitochondria and cytosol (Duchene et al., 2001), Golgi-like vesicles and cytosol (Rautengarten et al., 2011), plastids and endoplasmic reticulum (Levitan et al., 2005), and mitochondrion and endoplasmic reticulum (Lee et al., 2011). In addition, some proteins associate with the exterior of an organelle but do not penetrate the hydrophobic membrane and thus, whilst actually cytosolic, appear localized to a specific organelle (Rautengarten et al., 2011).

SUBA3 can assist by systematically searching for putatively dual-targeted proteins. Using the information from the prediction programs and the experimental data, lists of candidates for dual-targeted proteins can be generated. To compile a list of candidate proteins targeted to both plastid and mitochondrion, for example, SUBA3 can be queried for proteins that have been observed in these two locations by GFP tagging or mass spectrometry [(by “GFP” to be in “mitochondrion” OR by “MS/MS” to be in “mitochondrion”) AND (by “GFP” to be in “plastid” OR by “MS/MS” to be in “plastid”)]. This results in a list of 315 proteins that have been observed in both locations. Using SQL or manually analyzing the SUBA3 result file shows that in many of these cases (93 proteins) both locations are reported within the same publication. Therefore, these are known dual-targeted proteins. However, for over 200 proteins, the two locations were reported in different publications, and thus these are potentially unrecognized dual-targeted proteins. Repeating this process for plastids and peroxisomes gives a list of 86 proteins and for mitochondria and peroxisomes a list of 41 proteins. These potential examples of dual-targeting can be tested experimentally by GFP tagging, import studies or other approaches.

**Figure 2** shows that for three Deg proteases experimental localization data disagree. Both Deg1 and Deg9 have been localized to the plastid and nucleus using mass spectrometry (**Figure 2**). Similarly, Deg7 has been localized to the plastid by GFP tagging and to the cytosol by mass spectrometry (**Figure 2**). Searching GFP and MS data sets in SUBA using the procedure described above results in a list of 398 proteins localized to the plastid and nucleus and a list of 254 proteins for plastid and cytosol. Analyzing these two lists further shows that only four proteins for plastid/nucleus and twelve proteins for plastid/cytosol are reported by the same research group. Thus, for the majority of proteins the plastid/nucleus or plastid/cytosol dual locations were described in different publications, including Deg1, Deg7, and Deg9, and thus, these Deg proteases are potentially unrecognized dual-targeted proteins.

### EXPANDING DEG PROTEASE LOCALIZATION DATA FROM SUBA3 WITH NOVEL GFP TAGGING DATA

**FIGURE 3:**
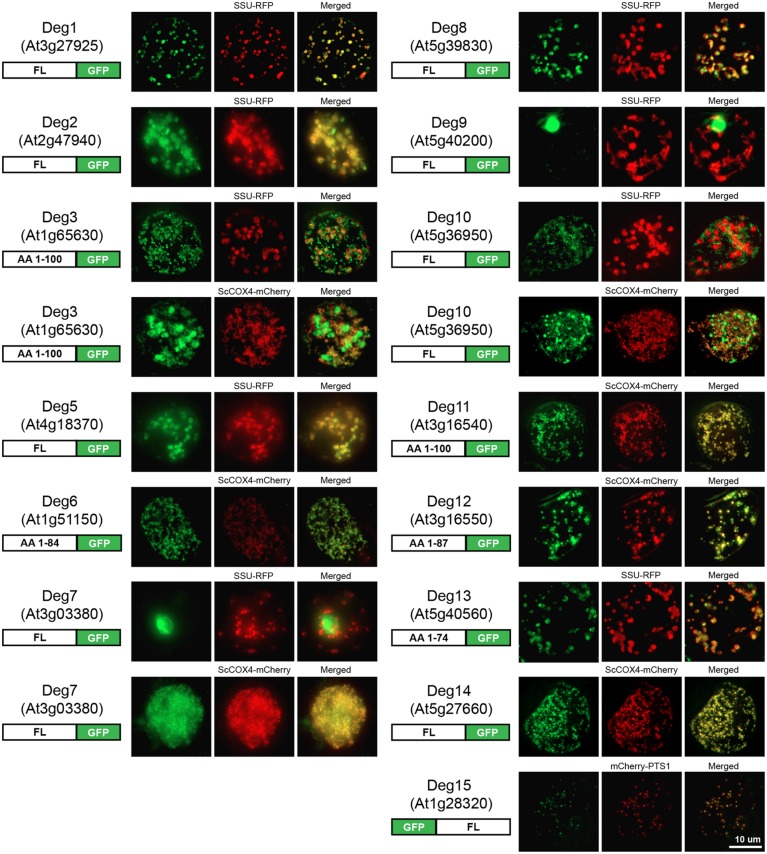
**Fluorescence images of the subcellular localization of Deg proteases by GFP tagging.** *N*- and/or *C*-terminal GFP fusion proteins were constructed. Either the full-length (FL) or part of the protein with the number of amino acids (AA) indicated in the schematic representation of each construct (left) were fused to GFP. Targeting ability was tested in *Arabidopsis* suspension cells using SSU-RFP as a marker for plastid targeting, ScCOX4-mCherry as a marker for mitochondrial targeting, and mCherry-PTS1 as a marker for peroxisome targeting. Multiple co-localizations are presented for Deg3, Deg7, and Deg10 as these Deg proteases localized to more than one subcellular compartment in this study suggesting dual-targeting or conflicting localization results. Scale as indicated.

When considering the Deg protease dataset as a whole, it became clear that the reasons for discrepancies could not be resolved without new data. The key experiments missing in order to determine if the proteomics data could be independently confirmed, and to resolve conflicts or multi-targeting, were to systematically fuse the 16 Deg proteases to GFP and observe localization by fluorescence microscopy. To do this, the full-length coding sequences were amplified from cDNA and used for the fusion with GFP. Due to the low expression of some of the Deg proteases or the possibility of being pseudogenes (Schuhmann and Adamska, 2012), their full-length coding sequence could not be amplified from cDNA. Where possible the first 300 bp were amplified from genomic DNA or, if the first exon was shorter than 300 bp, the longest possible region within the first exon was amplified (see schematic representations in **Figure 3**). These Deg sequences were *C*-terminally tagged with GFP as most of the Deg proteases were expected to be localized to the mitochondrion or plastid, based on prediction, and *N*-terminal fusions would have masked the targeting ability of the plastid and mitochondrial signal peptides. On the contrary, Deg15 contains a *C*-terminal peroxisomal targeting signal 1 (PTS1, Ser-Lys-Leu) and a *C*-terminally tagged Deg15 would be mistargeted. Thus, the GFP was fused to the *N*-terminus of its particular coding sequence. The data obtained are presented in **Figure 3** with additional co-localizations available in Supplemental Figure 1.

#### Plastid and mitochondrial Deg proteases

The plastid location of Deg1, Deg2, Deg5, and Deg8 reported by multiple proteomic studies was confirmed by GFP tagging (**Figure 3**). These proteases have been shown to function either in the chloroplast stroma (Deg2) or in the thylakoid lumen (Deg1, Deg5, Deg8) and it has been suggested that they are involved in the biogenesis of photosystem II, maintaining protein homeostasis in the thylakoid lumen, and degrading and repairing damaged proteins in the thylakoid lumen and the stroma (reviewed in Schuhmann and Adamska, 2012). The nuclear location of Deg1 found by MS studies could not be confirmed by GFP tagging, despite using a full-length fusion that should contain any nuclear localization signal of Deg1. The PPI data show that Deg1 interacts with TCP14 (At3g47620), a transcription factor localized to the nucleus that regulates seed germination and shows elevated expression level just prior to germination. The PPI study was a large high-throughput study based on the yeast-two hybrid system (Arabidopsis Interactome Mapping Consortium, 2011) and awaits independent confirmation. As we cannot exclude a location of Deg1 to the nucleus, perhaps during germination, this PPI data may be informative. However, both proteomics studies that found Deg1 localized to the nucleus were performed on adult plants so do not directly support the germination hypothesis.

The plastid location of Deg11 reported by a single proteomic study (Friso et al., 2004) could not be confirmed by GFP tagging. Instead Deg11 was found to be located in the mitochondrion (**Figure 3**). A mitochondrial location of this protease is also predicted by 14 of the 22 predictors in SUBA3, including TargetP (Emanuelsson et al., 2000), Predotar (Small et al., 2004), and YLoc (Briesemeister et al., 2010).

No experimental data on the location of Deg proteases Deg3, Deg6, Deg10, Deg12, Deg13, and Deg14 (**Figure 2** and **Table 2**) have been published, and nothing is known about their physiological roles. At least eight predictors in SUBA3 predict a mitochondrial location for Deg3, Deg6, Deg10, Deg12, and Deg14 and indeed we found these five proteases are targeted to the mitochondrion by our GFP tagging approach (**Figure 3**). Surprisingly, a fluorescence signal was also detected in the plastid when Deg3-GFP and Deg10-GFP fusion constructs were used to transform *Arabidopsis* suspension cells (**Figure 3**), suggesting that Deg3 and Deg10 are dual-targeted to the mitochondrion and to the plastid. Interestingly, Deg3 and Deg10 were also predicted to both the plastid and the mitochondrion when querying SUBA3 using Predotar, TargetP and WoLF PSORT [(“is” predicted by “Predotar” to be in “plastid” OR “is” predicted by “TargetP” to be in “plastid” OR “is” predicted by “WoLF PSORT” to be in “plastid”) AND (“is” predicted by “Predotar” to be in “mitochondrion” OR “is” predicted by “TargetP” to be in “mitochondrion” OR “is” predicted by “WoLF PSORT” to be in “mitochondrion”)]. Very recently, a semi-quantitative proteomic approach has detected Deg3 in low abundance in the stroma-lamellae of the thylakoid (Tomizioli et al., 2014) supporting a plastid location for this Deg protease without excluding a mitochondrial location. In addition, Deg13 was localized to the plastid in our study (**Figure 3**). Given these results we should bear in mind that the full-length coding sequence could only be amplified from cDNAs of Deg10 and Deg14. For Deg3, Deg6, Deg12, and Deg13 the first 300 bp or less were amplified from genomic DNA and fused to GFP. With proteomics data existing for Deg3 and Deg12 (Baerenfaller et al., 2008; Tomizioli et al., 2014), but transcript and protein data being absence from publicly accessible databases for Deg6 and Deg13, could indicate that these two Deg proteases are pseudogenes (Schuhmann and Adamska, 2012).

**Table 2:** Overview of the subcellular locations of Deg proteases in *Arabidopsis*.

AGI	Name	Locations SUBAcon	Locations previous GFP or MS experiments	Locations GFP tagging (this study)
At3g27925.1	Deg1	Plastid	Plastid, Nucleus	Plastid
At2g47940.1	Deg2	Plastid	Plastid	Plastid
At1g65630.1	Deg3	Mitochondrion	n.d.	Plastid, Mitochondrion
At1g65640.1	Deg4	Plasma membrane	n.d.	n.d.
At4g18370.1	Deg5	Plastid	Plastid	Plastid
At1g51150.1	Deg6	Plastid	n.d.	Mitochondrion
At3g03380.1	Deg7	Cytosol	Cytosol, Plastid	Nucleus, Mitochondrion
At5g39830.1	Deg8	Plastid	Plastid	Plastid
At5g40200.1	Deg9	Nucleus	Nucleus, Plastid	Nucleus
At5g36950.1	Deg10	Mitochondrion	n.d.	Plastid, Mitochondrion
At3g16540.1	Deg11	Mitochondrion	Plastid	Mitochondrion
At3g16550.1	Deg12	Mitochondrion	n.d.	Mitochondrion
At5g40560.1	Deg13	Plasma membrane, ER, Golgi	n.d.	Plastid
At5g27660.1	Deg14	Mitochondrion	n.d.	Mitochondrion
At1g28320.1	Deg15	Peroxisome	Peroxisome	Peroxisome
At5g54745.1	Deg16	Cytosol, Peroxisome	n.d.	n.d.

*Listed are the *Arabidopsis* gene identifiers (AGIs), the short names of the Deg proteases, the subcellular locations of the Deg proteases determined by SUBAcon, by GFP or mass spectrometry experiments published prior to this study, and by this study. ER, endoplasmic reticulum; n.d., location is not determined.*

#### Peroxisome Deg protease

The location of Deg15 in peroxisomes was previously demonstrated by both subcellular proteomic and GFP tagging studies (Eubel et al., 2008; Schuhmann et al., 2008). As shown in **Figure 3**, the peroxisomal location of Deg15 was confirmed again by this study. *In vivo* and *in vitro* analysis has shown that Deg15 is responsible for processing PTS2-containing proteins and plants lacking Deg15 display a phenotype potentially linked to reduced fatty acid β-oxidation due to lack of enzyme processing (Schuhmann et al., 2008).

#### Nuclear Deg protease

The SUBA3 query reported that Deg9 has been located by different proteomic studies to the plastid (Kleffmann et al., 2004) and to the nucleus (Pendle et al., 2005). The nuclear location of Deg9 could be confirmed here by GFP tagging (**Figure 3**). However, the plastid location could not be confirmed in this study. From the analysis of conflicts between published localization datasets (see Conflicts Between Published Localization Datasets) a nuclear location for Deg9 is also suggested to be more likely than a plastid location. As the physiological role of Deg9 remains to be elucidated, the possible plastid location of Deg9 will need to be proven by further independent analysis.

#### Deg7 protease

Deg7, the last of the proteases for which proteomic data are available in SUBA3, was reported to be located in the cytosol (Ito et al., 2011). Another study has found this protease in the chloroplast stroma by GFP tagging and immunoblotting (Sun et al., 2010). Neither of these two locations could be verified by GFP tagging in this study, instead Deg7 was localized to the nucleus and the mitochondrion (**Figure 3**). A nuclear location is plausible because Deg7 is putatively orthologous to the only fungal Deg protease, which is located in the nucleus (Schuhmann et al., 2011). The nuclear location of Deg7 could have been missed by Sun et al. (2010) as the authors fused only the *N*-terminal 243 amino acids of Deg7 to GFP, whereas in this study the full-length coding sequence of Deg7 was fused to the *N*-terminus of GFP. Interestingly, NLS Mapper (Kosugi et al., 2009) predicts a bipartite nuclear localization signal from 845 to 873 amino acids. The analysis of *deg7* mutant plants revealed that the mutant is more sensitive to high light stress than wild type as demonstrated by an inhibited growth phenotype of *deg7* mutants when exposed to high light. Additionally, it was shown that Deg7 interacts directly with PSII (Sun et al., 2010). Thus, a plastid location of this protease is likely and may have been missed in this study for a reason associated with the GFP fusion or the intensity of fluorescence in the plastid. Ito et al. (2011) localized Deg7 to the cytosol using a proteomic approach. Although purification techniques have improved immensely and the authors rigorously selected for cytosolic proteins, it is very difficult to avoid contamination of the cytosol by soluble proteins from organelles. Thus, the cytosolic location of Deg7 could be due to contamination during the cytosolic sample preparation. In fact, other soluble chloroplast proteins have been reported as cytosolic in this study, such as a chloroplast form of ATP sulfurylase (AT1G19920), a plastidic triose phosphate isomerase (AT2G21170), a chloroplast beta-amylase (AT3G23920), a plastidic uracil phosphoribosyltransferase (AT3G53900), and an *f*-type thioredoxin localized in the chloroplast stroma (AT5G16400). The localization of Deg7 represents an example of how contradictory location data can arise even when the same localization method was used.

#### Unknown location Deg proteases

Using GFP tagging we were unable to define a subcellular location for Deg4 and Deg16 (**Table 2**). Both proteases have been suggested to be potential pseudogenes, because no transcript or protein data could be found in publicly available databases (Schuhmann and Adamska, 2012). Indeed, we were only able to amplify the first 300 bp from genomic DNA of Deg4 and *Arabidopsis* suspension cells transformed with the Deg4-GFP fusion construct showed a GFP signal but the subcellular compartment could not be determined (Supplemental Figure 1).

## DISCUSSION

SUBA3 integrates protein localization information for Arabidopsis proteins from various sources, including data from bioinformatics prediction programs, from annotators and from experimental data sources. SUBAcon was developed to estimate a protein’s consensus location based on Bayesian probabilities calculated from all the experimental data and predictions available for each protein. By collating localizations from 22 different predictors and including experimental data as location evidence, SUBA3 overcomes the limitations of each of the individual predictors. In addition, SUBA3 allows the building of complex queries to investigate many different aspects of protein location and can be used effectively to select candidate lists of proteins for further experimental analysis as exemplified with the example of the Deg proteases presented here.

Searching SUBA3, no previous experimental evidence existed for eight of the 16 Deg proteases. This study systematically analyzed the subcellular localization of the Deg protease family and provided the first experimental evidence for six Deg proteases. Furthermore, it resulted in substantial experimental evidence for mitochondrial localized Deg proteases. Indeed, six Deg proteases were targeted to the mitochondrion by our GFP tagging approach, namely Deg3, Deg6, Deg10, Deg11, Deg12, and Deg14. Two of these, Deg3 and Deg10, additionally localized to the plastid, exposing novel dual-targeted Deg proteases in the plastid and the mitochondrion. In addition, Deg13 was localized to the plastid and the subcellular locations of Deg1 and Deg9 were further refined. Previously localized to both the plastid and nucleus, a sole plastid location was verified for Deg1, whereas a sole nuclear location was confirmed for Deg9. This study also confirmed previous results obtained for Deg2, Deg5, and Deg8 to be located in the plastid and a peroxisome location for Deg15, whereas contradictory results were obtained for Deg7.

The number of proteins for which localization information is available has increased dramatically over the last few years as well as the number of organelles for which at least part of the proteome is known. For example, the plastid proteome shows a 60% experimental coverage of the estimated plastid proteome size in *Arabidopsis* and seven Deg proteases were already experimentally located in plastids prior to this study (see Defining the Size of Experimental vs Predicted Datasets for a Specific Subcellular Location). However, this still leaves many more proteins to be discovered in this subcellular location as shown by the targeted localizations of Deg3, Deg10, and Deg13 to the plastid. Similarly, the experimental coverage of other subcellular locations is still limited (as indicated in **Table 1**) and there is room for many proteins to be experimentally identified in these locations even using available methodologies.

With the increase of localization information, the amount of contradictory information has also increased. For example, Deg9 has been localized by proteomic studies to the plastid and the nucleus by two independent research groups. Further analysis in SUBA3 suggests a nuclear location to be more likely than a plastid location (see Conflicts Between Published Localization Datasets). Accordingly, the nuclear location could be verified in this study, whereas the plastid location of Deg9 could not be confirmed.

Not only do different experimental approaches result in such discrepancies, even using the same approach by different researchers gives rise to different results as shown by the GFP localization of the Deg7 protease. Some of these discrepancies are yet to be recognized dual-targeted proteins. Thus, the identification of more multi-localized proteins (such as Deg3 and Deg10) is required to resolve these discrepancies. In addition, we need to find better ways of describing the net or final location of some proteins that may move around in the cell and are present for a time at various locations as in the case of proteins that enter the secretory system. However, there are real discrepancies and these will not be resolved by adding more and more of the same type of information. By adding useful additional experimental datasets, such as PPI data, and using predictions available for each protein, SUBAcon estimates a protein’s consensus location and also assists in finding missing organellar proteins. There is also published data from quantitative analysis of MS and GFP data that could be used as evidence for proteins not being in certain locations and this could be used to counteract more qualitative data from earlier published reports.

As seen from the example of the Deg protease family presented here, using SUBA to determine the subcellular location of members of a protein family can assist in defining the function of a particular protein family member. This gives insight into processes such as sub-functionalization, where gene families have expanded and then divided their functions between protein family members located in different parts of the cell. For example, bacterial genomes usually encode three Deg proteases, yeasts own one (occasionally duplicated), and four to five genes coding for Deg proteases are present in mammalian genomes (Kim and Kim, 2005; Rawlings et al., 2008; Huesgen et al., 2011). In plants, however, this family has expanded and plant genomes contain many more genes encoding Deg proteases. *Arabidopsis thaliana* possesses 16 Deg protease genes (Huesgen et al., 2005), *Oryza sativa* 15 (Tripathi and Sowdhamini, 2006), and *Populus trichocarpa* 20 (Garcia-Lorenzo et al., 2006). This relatively high number of Deg proteases in plants is mainly due to gene duplications (Schuhmann et al., 2012) and some additional Deg genes may result from a gene transfer from the plastid, which is of prokaryotic origin, to the nucleus. Combining subcellular location data in the future with sequence similarity information, evidence of more recent duplication events in genomes and synteny across related species will be needed to provide this insight systematically across gene families.

## AUTHOR CONTRIBUTIONS

Sandra K. Tanz collected the subcellular localization data from the literature, carried out the localization studies by GFP tagging, analyzed the data and drafted the manuscript. Ian Castleden developed the SUBA3 database and helped analyzing the data. Cornelia M. Hooper participated in collecting the subcellular localization data from the literature. Ian Small participated in the design of the study. A. Harvey Millar participated in the design of the study and helped to draft the manuscript. All authors read and approved the final manuscript.

## Conflict of Interest Statement

The authors declare that the research was conducted in the absence of any commercial or financial relationships that could be construed as a potential conflict of interest.

## Footnotes

1 http://suba.plantenergy.uwa.edu.au

2 www.ncbi.nlm.nih.gov/pubmed
