# Supplementary material for: Using the SUBcellular database for Arabidopsis proteins to localize the Deg protease family
Source: Front Plant Sci. 2014 Aug 12;5:396. doi: 10.3389/fpls.2014.00396 (PMC4130198; doi:10.3389/fpls.2014.00396)
Supplement: Supplementary file 1 [file Data_Sheet_1.ZIP › Supplemental Figure 1.PDF]

Supplemental Figure 1: A full set of fluorescence images of the subcellular localization of Deg proteases by GFP tagging.
N- and/or C-terminal GFP fusion proteins were constructed. Either the full-length (FL) or part of the protein with the number of amino acids (AA) indicated in the schematic representation of each construct (left) were fused to GFP. Targeting ability was tested in Arabidopsis suspension cells or onion epidermal cells using SSU-RFP as a marker for plastid targeting, ScCOX4-mCherry as a marker for mitochondrial targeting, and mCherry-PTS1 as a marker for peroxisome targeting. Scale as indicated.

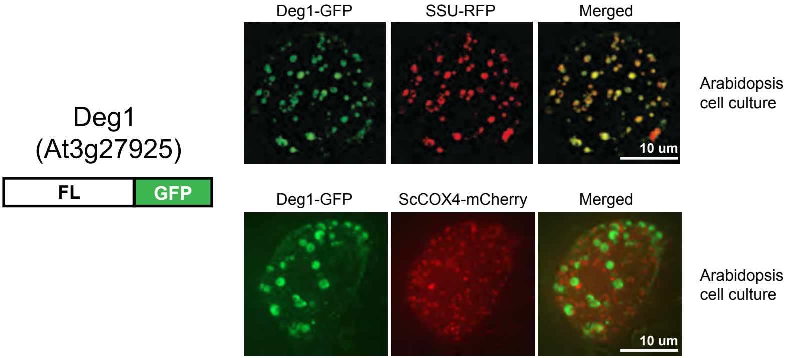

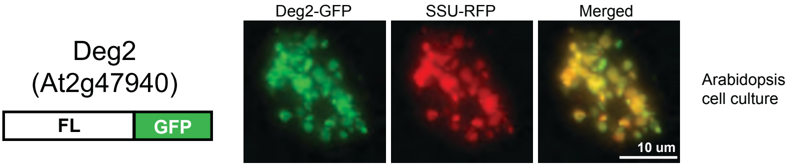
Deg2
(At2g47940)
FL
GFP
Deg2-GFP
SSU-RFP
Merged
10 um
Arabidopsis
cell culture

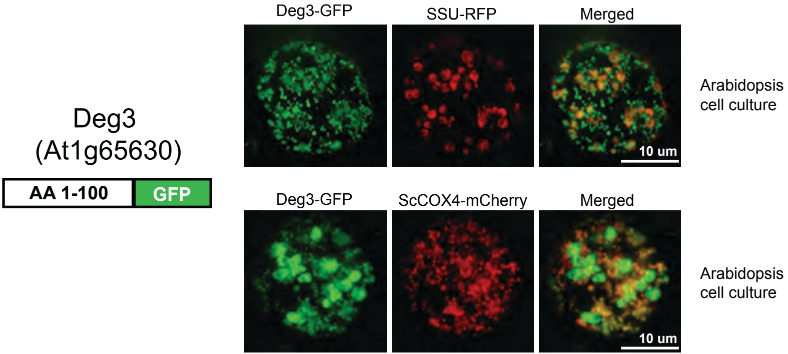
Deg3
(At1g65630)
AA 1-100
GFP
Deg3-GFP
SSU-RFP
Merged
10 um
Arabidopsis cell culture
Deg3-GFP
ScCOX4-mCherry
Merged
10 um
Arabidopsis cell culture

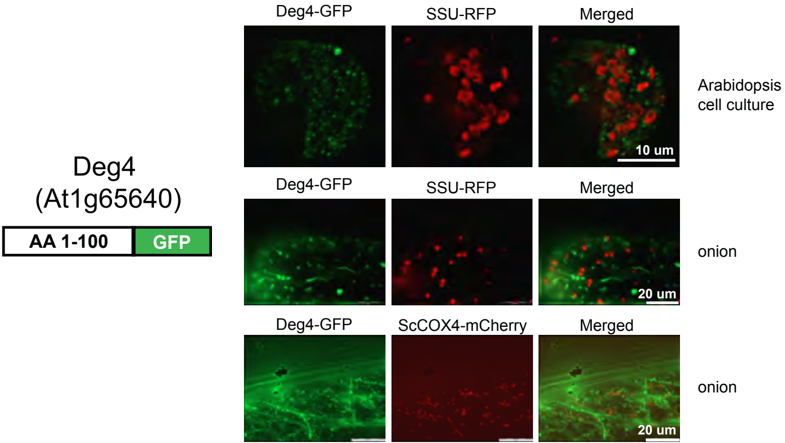
Deg4
(At1g65640)
AA 1-100
GFP
Deg4-GFP
SSU-RFP
Merged
10 um
Arabidopsis cell culture
Deg4-GFP
SSU-RFP
Merged
20 um
onion
Deg4-GFP
ScCOX4-mCherry
Merged
20 um
onion

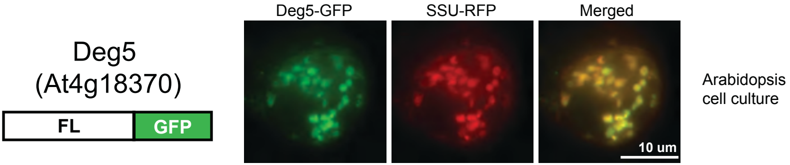
Deg5
(At4g18370)
FL
GFP
Deg5-GFP
SSU-RFP
Merged
10 um
Arabidopsis
cell culture

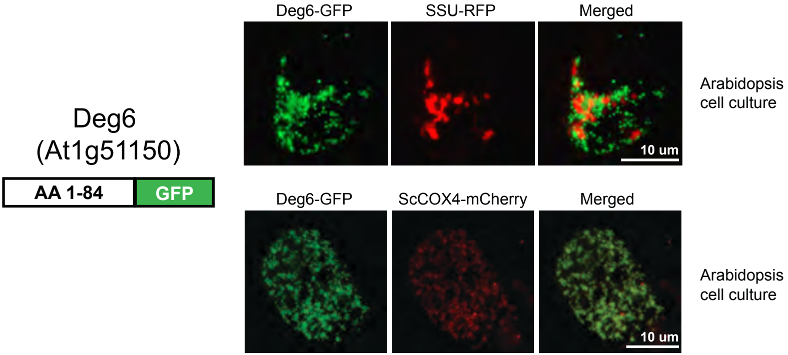

Deg6
(At1g51150)
AA 1-84
GFP
Deg6-GFP
SSU-RFP
Merged
10 um
Arabidopsis cell culture
Deg6-GFP
ScCOX4-mCherry
Merged
10 um
Arabidopsis cell culture

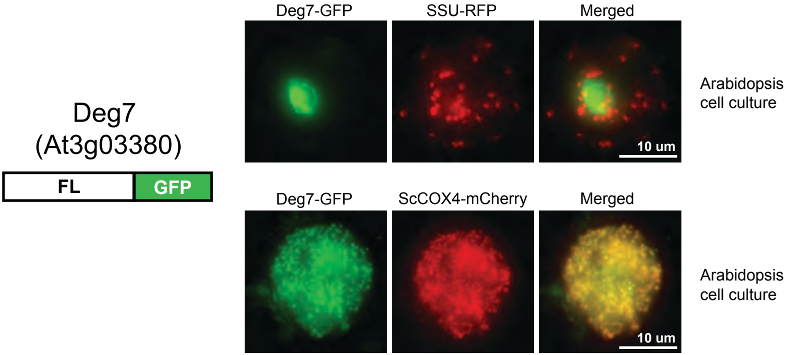
Deg7
(At3g03380)
FL
GFP
Deg7-GFP
SSU-RFP
Merged
10 um
Arabidopsis
cell culture
Deg7-GFP
ScCOX4-mCherry
Merged
10 um
Arabidopsis
cell culture

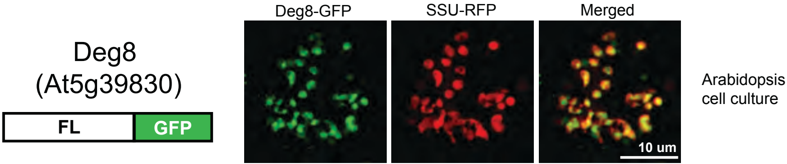

Deg8
(At5g39830)
FL
GFP
Deg8-GFP
SSU-RFP
Merged
10 um
Arabidopsis
cell culture

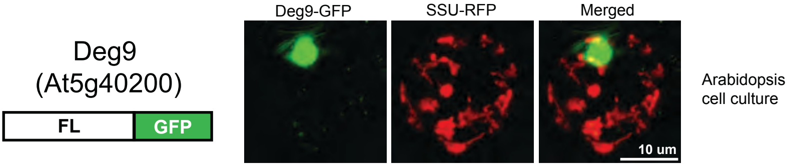
Deg9
(At5g40200)
FL
GFP
Deg9-GFP
SSU-RFP
Merged
10 um
Arabidopsis
cell culture

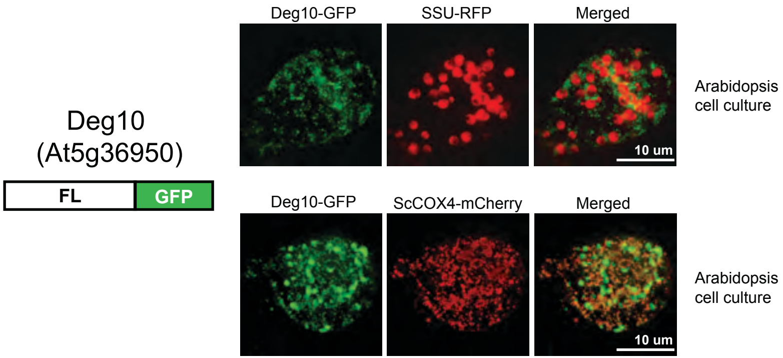
Deg10
(At5g36950)
FL
GFP
Deg10-GFP
SSU-RFP
Merged
10 um
Arabidopsis cell culture
Deg10-GFP
ScCOX4-mCherry
Merged
10 um
Arabidopsis cell culture

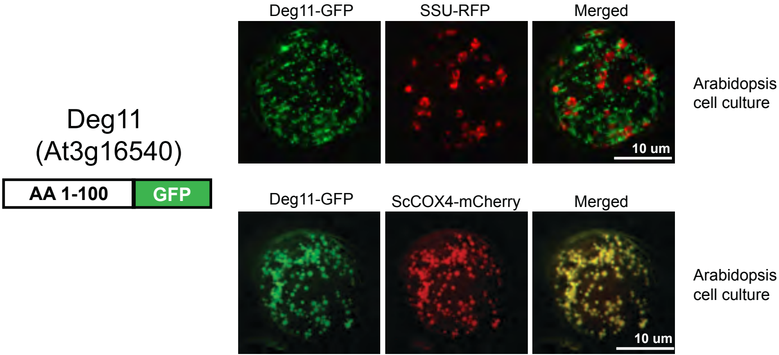
Deg11
(At3g16540)
AA 1-100
GFP
Deg11-GFP
SSU-RFP
Merged
10 um
Arabidopsis
cell culture
Deg11-GFP
ScCOX4-mCherry
Merged
10 um
Arabidopsis
cell culture

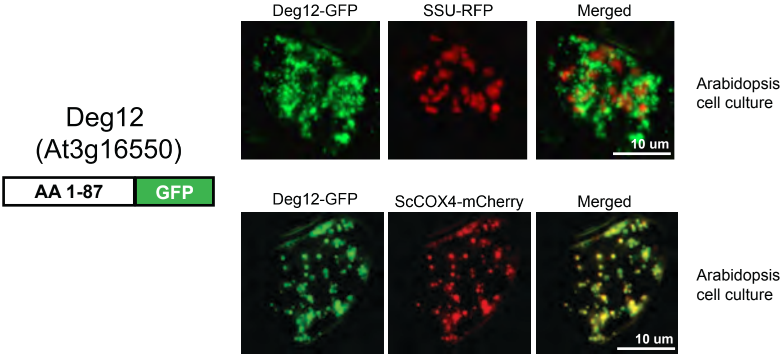
Deg12
(At3g16550)
AA 1-87
GFP
Deg12-GFP
SSU-RFP
Merged
10 um
Arabidopsis
cell culture
Deg12-GFP
ScCOX4-mCherry
Merged
10 um
Arabidopsis
cell culture

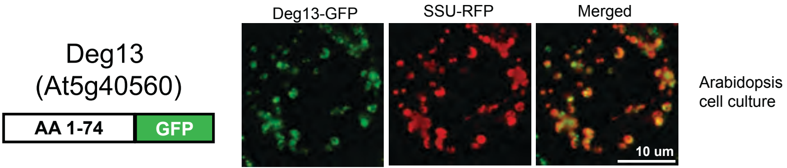
Deg13
(At5g40560)
AA 1-74
GFP
Deg13-GFP
SSU-RFP
Merged
10 um
Arabidopsis
cell culture

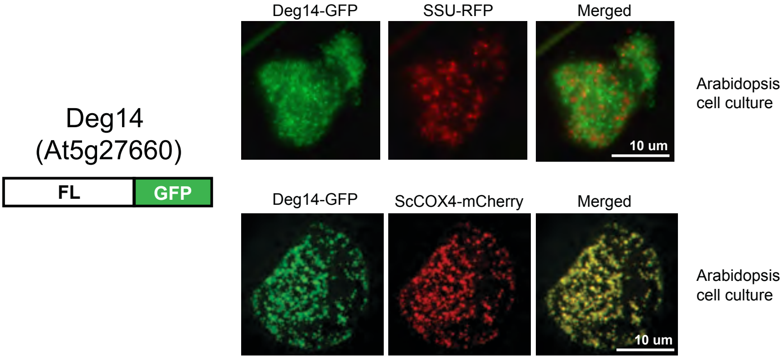

Deg14
(At5g27660)
FL
GFP
Deg14-GFP
SSU-RFP
Merged
10 um
Arabidopsis
cell culture
Deg14-GFP
ScCOX4-mCherry
Merged
10 um
Arabidopsis
cell culture

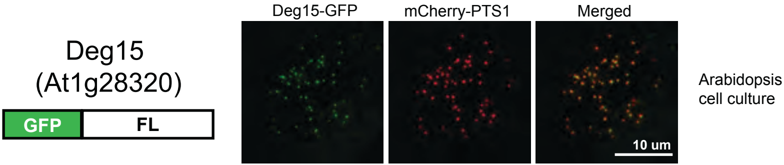

Deg15
(At1g28320)
GFP
FL
Deg15-GFP
mCherry-PTS1
Merged
10 um
Arabidopsis
cell culture
