# Supplementary material for: Using the SUBcellular database for Arabidopsis proteins to localize the Deg protease family
Source: Front Plant Sci. 2014 Aug 12;5:396. doi: 10.3389/fpls.2014.00396 (PMC4130198; doi:10.3389/fpls.2014.00396)
Supplement: Supplementary file 1 [file Data_Sheet_1.ZIP › Supplemental Table 1.PDF]

Supplemental Table 1: Primers used for Gateway® cloning. Either the full-length or the first 222-300 bp of the coding sequences of *Deg1* – *Deg16* were amplified with primers listed in this table containing the *attB* sites for Gateway® cloning.

**AGI**	**Gene name**	**5' or 3' primer**	**primer seq 5'->3' (w attB sites for Gateway cloning)**
At3g27925	*Deg1*	5'	GGGGACAAGTTTGTACAAAAAAGCAGGCTTCGAAGGAGATAGAACCATGGCGACGACGACGAGTTG
At3g27925	*Deg1*	3'	GGGGACCACTTTGTACAAGAAAGCTGGGTCTCCACCTCCGGATCCAGACTCGTCCGGCTTTGGTT
At2g47940	*Deg2*	5'	GGGGACAAGTTTGTACAAAAAAGCAGGCTTCGAAGGAGATAGAACCATGGCCGCCTCCGTAGCA
At2g47940	*Deg2*	3'	GGGGACCACTTTGTACAAGAAAGCTGGGTCTCCACCTCCGGATCMTGCCCACACCAGTCCATCAA
At1g65630	*Deg3*	5'	GGGGACAAGTTTGTACAAAAAAGCAGGCTTCGAAGGAGATAGAACCATGTCATTCCTCTGTGTTCGTAC
At1g65630	*Deg3*	3'	GGGGACCACTTTGTACAAGAAAGCTGGGTCTCCACCTCCGGATCCTACCTTCACCACCGAGTTGAG
At1g65640	*Deg4*	5'	GGGGACAAGTTTGTACAAAAAAGCAGGCTTCGAAGGAGATAGAACCATGTTGTTCCGATTTCTTCAAACAC
At1g65640	*Deg4*	3'	GGGGACCACTTTGTACAAGAAAGCTGGGTCTCCACCTCCGGATCCGCCGGATTCTTGCTGCGG
At4g18370	*Deg5*	5'	GGGGACAAGTTTGTACAAAAAAGCAGGCTTCGAAGGAGATAGAACCATGACCATGGCTCTTGCTTCTT
At4g18370	*Deg5*	3'	GGGGACCACTTTGTACAAGAAAGCTGGGTCTCCACCTCCGGATCMGAATCTGTCTCTATAAGCTGTTCC
At1g51150	*Deg6*	5'	GGGGACAAGTTTGTACAAAAAAGCAGGCTTCGAAGGAGATAGAACCATGTTGTTCCGGTCTGTTCATC
At1g51150	*Deg6*	3'	GGGGACCACTTTGTACAAGAAAGCTGGGTCTCCACCTCCGGATCCGCTTGAGTATTCTTTCTCAGTTGT
At3g03380	*Deg7*	5'	GGGGACAAGTTTGTACAAAAAAGCAGGCTTCGAAGGAGATAGAACCATGGGAGATCCGTTGGAGAG
At3g03380	*Deg7*	3'	GGGGACCACTTTGTACAAGAAAGCTGGGTCTCCACCTCCGGATCMCTGCAAGGCTTTCAATATATTTCTC
At5g39830	*Deg8*	5'	GGGGACAAGTTTGTACAAAAAAGCAGGCTTCGAAGGAGATAGAACCATGCAGGTAATAGCATCTTTCTGT
At5g39830	*Deg8*	3'	GGGGACCACTTTGTACAAGAAAGCTGGGTCTCCACCTCCGGATCCTGAACTTTTCTCTTCCAATGAGATC
At5g40200	*Deg9*	5'	GGGGACAAGTTTGTACAAAAAAGCAGGCTTCGAAGGAGATAGAACCATGAAGAATTCTGAGAAGAGAGGA
At5g40200	*Deg9*	3'	GGGGACCACTTTGTACAAGAAAGCTGGGTCTCCACCTCCGGATCMGTTTCTTTCTTCAGTCTTGAGATC
At5g36950	*Deg10*	5'	GGGGACAAGTTTGTACAAAAAAGCAGGCTTCGAAGGAGATAGAACCATGCTGCTCCGGTCATTTCG
At5g36950	*Deg10*	3'	GGGGACCACTTTGTACAAGAAAGCTGGGTCTCCACCTCCGGATCMAACCGCAGAACAAGAAGCCAAC
At3g16540	*Deg11*	5'	GGGGACAAGTTTGTACAAAAAAGCAGGCTTCGAAGGAGATAGAACCATGTTTTTCCGGCCGTGTGTTC
At3g16540	*Deg11*	3'	GGGGACCACTTTGTACAAGAAAGCTGGGTCTCCACCTCCGGATCCAGGATGACTTTCTTCTATTTTCTTCC
At3g16550	*Deg12*	5'	GGGGACAAGTTTGTACAAAAAAGCAGGCTTCGAAGGAGATAGAACCATGGTGTCCCGGTACTCGAG
At3g16550	*Deg12*	3'	GGGGACCACTTTGTACAAGAAAGCTGGGTCTCCACCTCCGGATCCGCCCCCATATGATTCTTGGTTA
At5g40560	*Deg13*	5'	GGGGACAAGTTTGTACAAAAAAGCAGGCTTCGAAGGAGATAGAACCATGATTATTACAAACGCTCATGTGG
At5g40560	*Deg13*	3'	GGGGACCACTTTGTACAAGAAAGCTGGGTCTCCACCTCCGGATCCTGGATAGCCAATAACATTTACTGATTC
At5g27660	*Deg14*	5'	GGGGACAAGTTTGTACAAAAAAGCAGGCTTCGAAGGAGATAGAACCATGATGAATTTTCTGAGAAGAGCTG
At5g27660	*Deg14*	3'	GGGGACCACTTTGTACAAGAAAGCTGGGTCTCCACCTCCGGATCMCATGTCTGGATTAGCCTCCTC
At1g28320	*Deg15*	5'	GGGGACAAGTTTGTACAAAAAAGCAGGCTTCGAAGGAGATAGAACCATGGATGTGTCTAAAGTTGTCAGC
At1g28320	*Deg15*	3'	GGGGACCACTTTGTACAAGAAAGCTGGGTCTCCACCTCCGGATCMTAACTTGCTAGGGATCACATCAC
At5g54745	*Deg16*	5'	GGGGACAAGTTTGTACAAAAAAGCAGGCTTCGAAGGAGATAGAACCATGAGTAATCAAGTAAATGATATATCTTATG
At5g54745	Deg16	3'	GGGGACCACTTTGTACAAGAAAGCTGGGTCTCCACCTCCGGATCMCTTGTCAGAAGGAGAATCATTTTC
At5g54745	Deg16	3'	GGGGACCACTTTGTACAAGAAAGCTGGGTCTCCACCTCCGGATCCTGCTCGTGGAGTTGCTGAAC
